# Care Ethics and the Future of Work: a Different Voice

**DOI:** 10.1007/s13347-022-00604-5

**Published:** 2023-01-26

**Authors:** Madelaine Ley

**Affiliations:** Ethics/Philosophy Section, Department of Values, Technology and Innovation, Faculty of Technology, Management and Policy, Delft University of Technology, Jaffalaan 5, 2628 BX Delft, Netherlands

**Keywords:** Care ethics, Future of work, Robot ethics, Embodiment, Emotions

## Abstract

The discourse on the future of work should learn from a turn in philosophy that occurred in the 1980s, one that recognizes the good life towards which ethics strives can only be reached on a foundation of caring relationships (Gillian, 1982; Noddings, 1984). Care ethics recognizes that human well-being is a group project, one that involves strong relationships, and concern for bodies and emotions. Too often, these features are left out of research exploring robotics in the workplace. This paper outlines the main tenets of care ethics, then applies the moral framework to the context of industrial and retail settings using robots. This approach sees these contexts through a relational lens, helping to identify, evaluate, and improve relationships critical to ensuring workers’ well-being. Second, care ethics considers taking care of people’s bodies beyond mere safety, examining how working with robots can exacerbate societal or economic pressures. Lastly, care ethics takes emotions as an important source of knowledge in building and supporting care. Additionally, this paper contributes to the care ethics literature by applying the framework to the context of robotized industrial workplaces, which has yet to be done.

## Introduction

This paper explores the future of robotized work through an uncommon lens: that of care ethics. In her book, *Moral Boundaries: A Political Argument for an Ethic of Care*, Tronto (1993 p. xi) offers care ethics not as a “complete alternative” to other moral theories, but rather “a glimpse into a different world, one where the daily caring of people is a valued premise of human existence.” For this paper, the words may be altered to a glimpse into a different world, one where the daily caring of people for each other is a valued premise of *human work*. From this perspective, the activities within robotized workplaces—namely factories, warehouses, and distribution centers—are not seen as mere tasks along a supply chain, but rather as points of connection between people. It is my contention that seeing through this relational lens and prioritizing caring connections in the workplace will have a profound impact on workers’ well-being.

Care ethics has been applied within the realm of technology and robot ethics before, focusing especially on the context of healthcare robotics (van Wynsberghe, 2016) and whether or not a robot has the ability to care (Sharkey, 2014; Sharkey & Sharkey, 2012). This paper departs from this literature by following a development in contemporary care research that sees care beyond typical contexts, like healthcare, and as a part of social infrastructure (Chatzidakis et al., 2020; Tronto 2013). It also aligns with robot ethicists who reject the position that robots are moral agents with the ability to care (Sharkey & Sharkey, 2012; van Wynsberghe and Robbins, 2019) and focus instead on how the machines can be integrated into a caring socio-technical system.^1^

Indeed, care ethics offers a unique perspective in the future of work debates by looking at how workers’ relationality, embodiment, and emotions are affected by robots in industrial and retail work settings. This approach sees these contexts through a relational lens, helping to identify, evaluate, and improve relationships critical to ensuring workers’ well-being. Second, care ethics considers taking care of people’s bodies beyond mere safety, examining how societal or economic pressures are taken up in an embodied way. Lastly, care ethics takes emotions as an important source of knowledge in building and supporting care. Additionally, this paper contributes to the care ethics literature by applying the framework to the context of robotized industrial workplaces, which has yet to be done.

The paper is structured as follows: Section 2 presents four different perspectives on the future of work, emphasizing how workers’ experiences should receive more critical attention. Section 3 introduces care ethics as a valuable tool for understanding the effects of robots at work and outlines the main features of the moral framework. Finally, Section 4 applies the lens of care ethics to the context of industrial and retail workplaces that use robots, suggesting how these machines may affect people’s relationships, including embodied and emotional relationships. Despite the paper’s tidy structure, however, I remind throughout that taking up an ethic of care in practice is rarely so well-organized. Care activities are often determined in the moment according to contextual particularities. This can be frustrating in cultures that value generalizable principles and checklists. Nonetheless, I invite the reader to open themselves to the messy tangle of caring. It is often unequal, always ongoing, and absolutely pivotal to our well-being.

## Part One: Robots at Work: Disconnected Narratives

This section provides an overview of the common narratives in the future of work discourse by drawing from a range of sources, including technical research, press releases, academic economics and philosophy, and news media. Workers’ voices show that technical research, corporate messaging, and academic debates are often disconnected from the stress and hardship of those working with robots. I argue for a different voice in ethics research, one that grapples with the experience of relationships within robotized workplaces.

### The Technical Aspects

I use the term “robot” to refer to “an autonomous system which exists in the physical world, can sense its environment, and can act on it to achieve some goals” (Mataric, 2007 p. 2), and focus on those used in industrial settings or stages along the retail supply chain. Much of the activity in these settings is repetitive and predictable, and the physical environments are structured, which lends them to robotization. Robots are already used in factories, distribution centers, and stores, ranging from heavy lifting to transporting goods to scanning shelves. The range of robotization varies in each context; for example, some Amazon distribution centers have robots transporting goods across the work floor (“What robots do [and don’t do] at Amazon fulfillment centers”) and some of Ahold Delhaize’s distribution centers are almost entirely automated (“‘Distribution centre of the future’ for Ahold Delhaize” 2018).

The next wave of robots in industry and retail differs from the ancestors found in factories behind safety cages (Fletcher & Webb, 2017). They are dynamic, lightweight, and adaptable (Gilchrist, 2016). Instead of being designed to avoid contact with workers or to stop immediately when contact is made, these robots are made to move among human workers, either taking over certain tasks in the division of labor and/or working in side-by-side collaboration (Bendel, 2018). Research in Industry 4.0 and 5.0 further integrate humans and robots by developing the latter as tools of enhancement and augmentation (e.g., exoskeletons) (Longo, Padovano and Umbrello 2020). All of these robots are made possible by technical advances that allow robots to sense and adapt more efficiently to abrupt or unexpected events in their environment. They also shape a new kind of relationship between humans and robots, one that is defined by fluidity, close proximity, and mutual attunement.

### The Visions of Industry

Companies using robots rely upon the oft-used narrative that a new technology will alleviate the burden of labor and allow more time for fulfilling work. For example, Walmart press releases claim that the shelf-canning robot “Freddy” helps employees endure “less drudgery and enjoy more satisfying jobs” (Harwell, 2019) and Amazon claims that in their warehouses “humans and robots work harmoniously to get packages to customers on time” (“What robots do [and don’t do] at Amazon fulfillment centers”). This message is echoed by international consulting firms, like Deloitte, whose consultants write: “The broader aim is not just to eliminate routine tasks and cut costs, but to create value for customers and meaningful work for people” (2019). These visions depict smooth worker-robot cooperation, an efficient supply chain, and satisfied customers.

### The Academic Research

Massive job loss and unemployment have been a main concern in the discourse on robotics and AI (Frey & Osborne, 2017), prompting debates on political solutions like universal-basic income (Vermeulen et al., 2020) and philosophical arguments for the value of a work-free life (Danaher 2019). However, recent research has shown that automation, robotics, and AI will likely not cause a total loss of jobs, but rather a reorganization of labor (Autor, 2014; Brynjolfsson & McAfee, 2016; Went et al., 2015; Willcocks, 2020).

Navigating the reorganization generally leads to a discussion on what work humans should do and what should be left to robots. The latter is often handed the so-called boring repetitive tasks so that humans have “more opportunity for the creative and interactive work” (Brynjolfsson & McAfee, 2016, p. 166). This allocation of tasks counters the concern that robots will take over jobs, envisioning a more cooperative and collaborative dynamic between humans and machines. History shows, however, that technology does not necessarily ease the lives of workers and can even add new burdens and more work (Cowan, 1983).

While there may be clear benefits of using robots for things like heavy lifting or entering dangerous situations, a critical eye must be kept on how and why tasks get defined as human or robot work, and whether the outcome is really less burdensome for human workers. According to the Moravec paradox in AI and robotics, what humans can do easily is very difficult to get robots to do (e.g., picking up a glass) and sometimes what humans find difficult is easy to have robots do (e.g., repetitive tasks for many hours or analyzing massive data sets) (Moravec, 1988). Therefore, what gets deemed “human work” may be based on the technical limitations of robotics rather than a normative stance on what is good for people to spend their time doing. For now, what robots can do and what some claim they *should* do tend to be conflated because they are aligned at a technical level. But the descriptive and the normative will have to be untangled as technical capacities advance, and explicit decisions will have to be made about what robots should and should not do.

The literature in Industry 4.0 and Industry 5.0 shifts focus from task allocation and co-working to human enhancement and augmentation through the use of robotic tools (Longo, Padovano and Umbrello 2020). Here, the robot is not seen as a replacement or a separate co-worker, but as something to incorporate unto the body (e.g., exoskeletons) or into the workplace system (e.g., robotic arms for heavy lifting) for increased capabilities and safety. Whether researchers see robots as tools or as co-workers, there is general agreement that a clear hierarchy of humans over machines should be maintained and that the use of robots should lead to less burdensome and more meaningful work. Though sociality and relationality are highlighted in the literature on meaningful work (Smids, Nyholm & Berker, 2020), there is an underlying individualism, which maintains the focus on how individual workers can pursue meaningfulness on the job.

There are recent explorations into the feelings of industrial or retail workers as they are placed beside robots. Fletcher and Webb (2017) consider the potential for psychological harm in industrial spaces deploying dynamic robots, suggesting that asking employees to work beside robots after years of being told the machines were fatally dangerous could lead to anxiety. van Wynsberghe et al. (2021) expand the concept of psychological harm to include potential emotional harm caused by the sense of working alongside one’s replacement and/or being surveilled. van Wynserghe et al.’s paper builds on broader insights from the contemporary philosophy of technology that sees technology as value-laden (Friedman & Hendry, 2019; van de Poel, 2013) and something humans both shape and are shaped by (Idhe, 2002; Verbeek, 2006). In this field, others have discussed how oppressive power dynamics can be diminished or exacerbated through the use of robotic technologies (Bryson, 2010; Coeckelbergh, 2021; Nyholm, 2020).

None of this research, however, has discussed how caring relationships, which involve the body and emotions, are vital in shaping well-being at work with robots (discussed in Section 3).

### The Workers’ Voices

While robots in the workplace might offer relief from heavy lifting or repetitive tasks, listening to workers’ voices shows that the technology is not necessarily liberating. Employees in Amazon distribution centers refer to their work alongside robots as a “cyborg job” and compare themselves to the machines, saying that as a worker you need to “clamp down on your self-respect and dignity” and “learn how to get harder and more pragmatic… Like a robot” (Guendelsberger, 2019). Reports from these warehouses also tell that some workers wear diapers in an effort to keep up with their robotic counterparts, often ignoring pressing pain and injuries (Guendelsberger, 2019). After the introduction of the robot in Walmart, employees reported that they adapted their behavior to become more robotic and machine-like, reporting feeling that the company did not value their work and that they have “never felt more robotic” (Harwell, 2019). These two corporations, known for maintaining efficiency along a vast supply chain, may seem extreme examples but workers across cultures and industries report that the presence of robots increases stress around job insecurity, burnout, and worry around adequate training with robotic technology (Yam et al., 2022).

It would be correct to argue that this is a problem of increased automation in general, insufficient labor laws, and a careless culture that demands fast delivery at the cost of human dignity. That being said, I believe that robots deserve particular attention as they can exacerbate anxieties and pressures on workers when not designed or deployed with care or into a caring infrastructure. It is only a piece of a larger puzzle, but nonetheless important to understand how well-being is either cared for or neglected when robots are introduced to the workplace.

## Part Two: A Different Voice

Discourse on the future of work is not (but should be) taking advantage of a development within Western philosophy, namely that of care ethics. Since the 1980s, the theory has had wide-ranging applications, from health care (van Wynsberghe, 2016) to democratic process (Tronto, 2013) to economic policy (Nelson, 2011). Despite the variation, care ethicists commonly challenge the culture’s proclivity towards individuality and unconstrained economic growth, arguing for a shift into a politics of interdependence that prioritizes well-being over profit (Chatzidakis et al., 2020; Tronto, 1993 & 2013). Focus on care (and carelessness) should be turned to the context of robotized workplaces, as cost-efficiency and optimizing supply chains risk taking priority over the well-being of those working alongside robots.

Within the field of robot ethics, discussion of care is often relegated to the contexts of home (Sorell and Draper, 2014), elderly care (Sharkey & Sharkey, 2012), and child education (Sharkey, 2016; Tanaka & Kimura, 2009). Care ethics as an approach has been applied in medical robots (van Wynersberghe 2013), service robots (van Wynersberghe 2021), facial recognition algorithms (Asaro 2020), and engineering education (Russel and Vinsel 2019). But it has not yet been applied to the future of work with robots in general, nor to the particular context of industrial or retail sectors. At first glance, distribution centers or factories may seem an unlikely place to make use of care ethics, but I argue that the framework is needed anywhere human well-being is at stake. As made clear in reports from the last few years, a robotized workplace is certainly such a place. The objectification and dehumanization of industrial or retail workers are escalated by increasingly formalized and digitized methods of optimization (van Wynsberghe, Ley & Roeser, 2021).

In the following section, I explain the fundamental commitments made by care ethicists and outline contemporary articulations of the framework before developing my own iteration for industrial or retail work with robots.

### Origins and Foundational Commitments

Care ethics originated in the 1980s with Carol Gilligan challenging her supervisor, Lawrence Kohlberg. She argued that girls’ concern for close relationships and how others feel when making moral decisions did not indicate immaturity, as Kohlberg concluded, but revealed a “different voice” from the dominant (male) concern with individualism and autonomy (1982). This voice had been historically overlooked in research on moral development, despite its prevalence in daily life. Following Gilligan’s seminal work, *In a Different Voice*, care ethics has been taken up by others as an alternative moral theory positing that relationships of care are the basis upon which life and flourishing arise.

Care can be defined in various ways, but a commonly accepted articulation is the following by Joan Tronto and Bernice Fisher:

a species activity that includes everything that we do to maintain, continue, and repair our ‘world’ so that we can live in it as well as possible. That world includes our bodies, our selves, and our environment, all of which we seek to interweave in a complex, life-sustaining web (1993, p. 103)

Tronto further argues that care must be seen as more than an attitudinal stance towards someone or something and instead understood as a practice that involves “both thought and action” (1993, p. 108). It is an ethics based on connection, of “taking the other’s needs as a starting point for what must be done” (Tronto, 1993, p. 105). Virginia Held reminds that discerning these needs “involves attention, empathic response, and a commitment to respond to legitimate needs’’ (Noddings, 2010). It is through these kinds of committed relationships that humans not only survive, but also come to enjoy a sense of well-being.

While different versions exist, there are commonly held beliefs that form the foundation of care ethics philosophy (Gilligan, 1982; Hamington, 2004; Held, 2006; Noddings, 1984; Tronto, 1993 & 2013). First, care ethics sees human interdependence as a given part of existence, thereby challenging dominant political and ethical theories that prioritize individualism (Tronto, 1993, p. 101). To iterate this point, Held provides a blunt debunking of the Hobbesian state of nature: “The picture represents persons as having sprung from nowhere like mushrooms (which was Hobbes’s own metaphor) with no notice of persons having been born of mothers and having received a huge amount of care before attaining whatever measure of independence they have” (Held, 2011). Care ethics reminds that humans cannot even be born on their own, let alone survive after that. Simply put, our need for others is primordial and does not fade over time.

Second, vulnerability or weakness is not something to shy away from, but is a central aspect of human beings that should be recognized in each person (Hamington, 2004; Tronto, 2013). In this sense, care ethicists often explicitly reject the neoliberal ideology and policy that emerged in the 1980s and sought to shift responsibility of care further from the state and unto individuals. Under this prevailing politic, “dependence on care has been pathologised, rather than recognized as a part of our human condition” (Chatzidakis et al., 2020, p. 23). The denial of humans’ innate need for others within neoliberalism is deeply intertwined with moralization of wealth and poverty, where financial independence is the golden standard and financial dependence is indicative of moral deficiency (Eubanks, 2017). The result is a “callous and uncaring climate for everyone” (Chatzidakis et al., 2020 p. 13). An ethics of care sees each person as innately valuable, despite their income or external successes. The tendency to evaluate people on their income or employment is important to keep an eye on when discussing the future of work, as lower-wage jobs can be seen as disposable or unimportant. Interestingly, the recent COVID-19 crisis has called this ranking of jobs into question as the traditionally undervalued work was shown to be essential. Still, these workers are often underpaid and overworked.

Lastly, care ethics is always entangled with the body and emotions. Care ethicists remind that the good life, towards which ethics aims, relies on the support and nurturing of people’s bodies (Hamington, 2004; Held, 1984). The fact that we are born as vulnerable bodies, unable to care for ourselves, signifies our relational ontology: there is no being without one another. Though many strive for independence as they age, bodies always slip into illness, injury, or depletion and at some point will require another’s help. At the same time, bodies are what we care about (Hamington 2004). Hands hold, ears listen, eyes observe, and mouths discuss. Caring is often directed towards the maintenance and support of bodies, which in turn enables people to take up the embodied practice of care.

From Gilligan’s study on girls’ moral decision-making onwards, care ethicists have argued that emotions are a driving force in shaping people’s lives and relationships (Gilligan, 1984; Pulcini 2016). Shifting from a history of philosophy and ethics that deemed emotions irrational or weak, an ethics of care regards emotions as critical motivators for action. Love may bind parents to their children, anger may spur people into revolt. While Tronto points out that emotional connection is not necessary for caring (e.g., a nurse is not emotionally affected by each patient), emotions nonetheless often play a hand in motivating people to care (1993). Furthermore, in order to care well for another, it is necessary to understand their needs and this requires understanding how they are feeling. For example, feeding a child and providing them shelter while ignoring their fears and confusions is not enough to care well.

### Contemporary Variations

Increasingly care ethics literature argues that care should not only be prioritized at the individual and community level, but should also be at the level of government (Chatzidakis et al., 2020; Tronto, 2013) and business (Hamington & Sander-Straudt, 2011). A number of recent policy reports have argued for the central place of care in society and the economy, including Oxfam’s *Time to Care* report (Lawson et al., 2020), the Women’s Budget Group’s *Creating a Caring Economy: A Call to Action* (2019); and the Leap Manifesto’s progressive climate action plan (2017). Each of these reports highlights the need to recognize and support the unpaid care work that sustains human life, and calls for a reprioritization of values such that human flourishing ranks higher than profit. In this sense, care ethics is at once a narrow focus on the nitty–gritty things that keep human life going *and* a large-scale political critique of the neoliberal status quo. I will argue below that the two levels should be understood together when considering how workers are affected by the introduction of robots.

A care ethics approach has also been developed within the field of robot ethics (van Wynsebrghe, 2013). Care-centered value-sensitive design (CCVSD) is an adaptation of value-sensitive design (VSD) that provides a normative grounding to guide the evaluation and design of robots according to care ethics. A fundamental tenet of VSD is that technologies are not objects in the world without value, but are manifestations of the value choices made by the person, team, and company that developed them (Friedman & Hendry, 2019). Building on Tronto’s work, CCVSD provides a framework so that the values of attentiveness, responsibility, competency, and responsiveness can guide the evaluation and design of robots (Van Wynsberghe, 2013).

van Wynsberghe’s CCVSD makes major strides in translating care ethics into a practical framework and has subsequently been taken up by other technology researchers focused mostly on care robots **(**Poulsen & Burmeister, 2019; Umbrello et al., 2021). However, CCVSD draws on a specific selection of care ethics literature with the explicate intention to create a design methodology, often with a focus on care robots. My intention in this paper is to provide a broader theoretical argument for applying care ethics in the discourse on robots at work, and the insights provided here might be useful in the application of CCVSD. In the following section, I show how the care ethics lens draws attention to the relationships, both embodied and emotional, that are critical for promoting well-being at work.

## Part Three: Care Ethics and the Future of Work with Robots

The care ethics approach I propose in this paper follows the fundamental commitments mentioned above—namely that humans are interconnected beings, each person is vulnerable and valuable, and our well-being depends on one another. Following contemporary iterations, I see care not only in the direct interactions humans have with one another, but also in the infrastructure societies set up to maintain the basic needs and well-being of its people. While care ethics does discuss both technology and embodiment, it does not come equipped with a deep account of either, so I draw on robot ethics (Coeckelbergh, 2021; Dobrosovestnova & Hannibal, 2020; Nyholm, 2020; van Wynsberghe, 2013), philosophy of technology (Pesch & Roeser, 2015), and feminist phenomenology (Young, 1980) to further conceptualize the impact of robots in the workplace.

### Relationships as the Core (Way of Seeing)

Research on the future of work generally discusses three main stakeholders: the engineers developing technologies, the companies deploying it, and the workers using it. This may be a useful shorthand, but the reality is a more varied and complex network that makes robotization possible. The range includes, but is not limited to the following: frontline workers, managers, maintenance workers, engineers, technical and non-technical researchers, manufacturers, project managers, project owners, consultants, business accountants, sales representatives, lawyers, trade unions representatives, lobbyists, media outlets, and politicians.^2^

It is the interactions between all of these people that make up the current and future robotization of work. Too often, however, research focuses narrowly on the human–robot interaction (HRI) that occurs between worker and machine. HRI studies can tell a lot about how people feel around robots (Müller-Abdelrazeq, Schönefeld, Haberstroh and Hees 2019), how robots and humans can communicate (Bonarini 2020; Gleeson et al. 2013), or how a robot can learn in its environment. However, there has been a recent call for a “paradigm shift” in the field of robot ethics to expand the focus beyond traditional HRI to human–robot-systems interaction (HRSI) (van Wynsberghe & Li, 2019). The argument is that by honing in on the relationship between humans and robots, the HRI model misses the effects and ethical concerns that occur beyond the dyad (2019, p. 17). When introduced into a particular context, the HRSI model looks not only at how a robot and person engage with one another, but also at the robot’s indirect affects. In a medical setting, for example, the robot has a direct impact on the patient it interacts with, but also on the schedules of hospital staff and frequency of appointments with patients (van Wynsberghe & Li, 2019, p. 17).

The HRSI model was originally applied in the context of health care and has since been extended to industrial and retail work (van Wynsberghe, Ley and Roeser 2021). Using the HRSI perspective with a care ethics approach helps to consider relationships beyond the human–robot dyad, but more needs to be done in order to identify the relationships workers have occurring at multiple levels. When a robot is introduced at work, one’s immediate, organizational, and societal relationships shift. The first is characterized by temporal and physical immediacy, including the direct and daily interactions people have with one another. The use of a robot at work can alter these interactions, for example, by spreading workers out so that they are not able to chat easily.

Organizational relationships are less direct, referring to how people relate to the organization they work for. A person’s sense of belongingness, security, and value in their employment will shape this relationship, as will the institutional makeup of the company. For example, if workers are given a safe space to voice their feelings about working with robots and the company adapts to the mentioned concerns, a relationship of trust may form despite the disruption that can occur in integrating robots.

Lastly, workers’ relationships may also shift at a societal level. Increased automation may alter how a person relates to their government, to political parties, or to other social groups within their culture. At this level, relationships are affected by how robots are depicted in the news and media, the way robotization of the industry is treated politically (e.g., economic strategy or workers’ rights policies), or the way different types of work are valued in a society. An example of this would be when a worker anticipates increased robotization at their place of work and they switch political allegiances to support a party that protects their employment.

Distinguishing immediate, organizational, and societal relationships helps to see how robots can cause relational shifts at various levels, but these categories should not be understood in isolation. On the contrary, they often coexist and shape one another. For example, a worker might enjoy the intuitive and competent design of a new robot at work, which makes them feel taken care of by the company they work for and supportive of a political party that supports advancing automation. At the same time, they may have been more accepting of the robot because they already have a positive relationship with the company they work for and generally lean towards technologically progressive visions of the future.

This section has looked at work through a relational lens, and in the next section, I will elaborate on how relationships across the levels can be more or less caring. First, however, I want to note two upshots about taking a relational understanding to work. One, in recognizing the multiple levels of relationships that make up a person’s experience of work, there is a move away from the bootstrap mentality that says an individual is solely responsible for his or her employment successes and well-being. Instead, a person’s well-being at work is made up of his or her own character and skills, but also of how they are cared for through the institutional setup and political context. Responsibility for worker well-being is partly lifted from the shoulders of individuals and distributed throughout the network of people involved in robotics at work. How exactly responsibility is allocated is beyond the scope of this paper, but it is important to highlight that a care ethics perspective makes a foundational shift from individual to collective responsibility for well-being.

### Mapping Caring Relationships

Identifying relationships is an important step, but only the first. To move from the merely descriptive and into the normative realm, the question arises: what makes a *good* relationship according to care ethics?

The theory brings reciprocity to the fore, which does not mean equal input or sameness, but rather that each party is encouraged to engage in a way that they are capable of and is contextually appropriate. Often, caring relationships will have an asymmetrical dynamic. The key is that there is mutual involvement, where decision-making and action are discerned through a process of mutual discernment and attunement. Tronto’s work here is helpful, as she breaks down five necessary elements of the caring relationship. First, there needs to be attentiveness, wherein someone suspends their preconceived notions in an effort to discern the needs of another (Tronto, 1993, p. 127). Second, there must be a sense of responsibility that pulls one to act upon the previously assessed need(s) (Ibid. p. 131). Third, there needs to be competence in the act of caregiving, which prevents unhelpful charity and inactive sentiment from being considered care (Ibid. p. 133). Fourth, there needs to be responsiveness from the care receiver, which empowers them to participate in how they are cared for (Ibid. p. 136). Lastly, in her more recent work, Tronto adds solidarity to the list, which requires that “caring needs and the ways in which they are met need to be consistent with democratic commitments of justice, equality and freedom for all” (Tronto 2013).

After identifying the multi-level relationships (Section 4.1) that exist in a work context, they might then be assessed according to Tronto’s five normative elements of care. To do so in the context of robotics, one might apply an updated version of van Wynsberghe’s CCVSD (2013) so that solidarity is included in the analysis. Using Tronto’s five features is helpful alongside mapping out relationships because it provides a more systematic way of evaluating these connections. However, further research into the application of care ethics in robotic environments would inspire alternative methods of assessing care relations and gaps.

### Care Gaps

Some relationships in the workplace may be clearly caring as they involve the features of care outlined by care ethicists like attentiveness, responsibility, mutual involvement, and empathy (Hamington, 2004; Noddings, 2010; Tronto, 1993). Others may be less caring, by lacking one or more of these elements. The latter case can be understood as a “care gap,” and once identified can be improved upon to foster a more caring connection. Care gaps—those relationships not involving sufficient attentiveness, responsibility, competence, responsiveness, and solidarity—are not uncommon nor unique to robotics in the workplace. However, robots being introduced or increased in a workplace can affect care gaps in one of three ways: existing gaps may be exacerbated, existing caring relationships may be weakened, and new relationships may emerge that include a care gap.

In the first case, consider companies that already engage in “care washing,” a term the Care Collective uses to describe “corporations trying to increase their legitimacy by presenting themselves as socially responsible ‘citizens’, while really contributing to inequality and ecological destruction” (2020, 12). Despite using the language of care, there may not be sufficient action. Or, if there is action, it may be taken without any having discerned the legitimate needs of those supposedly cared for. When robots are introduced or increased in a workplace, there may be (often public) pronouncements that workers will be taken care of, yet without action to understand what this entails and taking up responsibility for it, employees can be left even more vulnerable than before.

The second way robots may affect care gaps is by weakening a previously existing caring relationship. An example of this might be between a manager and frontline workers, who once shared a lot of face-to-face contact. A new robot might distance the two, leaving the manager to learn about workers more through the data collected about workflow, rather than conversation and in-person meetings. While data analysis might bring to light insights to increase the efficiency of work, it cannot tell managers how people are feeling or aching or finding meaning in their work. An over-reliance on data can limit a manager’s ability to be attentive to the full needs of workers and can leave workers with a sense that they cannot voice their needs.

The third way care gaps occur is within new relationships that emerge. An example might be the new connections between those building a robot and those working with it. While engineers may feel a responsibility towards end-users and they may have the potential to competently develop the technology so that it meets the needs of workers, if they do not also take the time to learn from the workers as well as incorporate their insights and needs in the technology development, this relationship cannot be considered one of care.

Care ethicists recognize that not all relationships need to be equally close, asymmetries will exist, and some connections will be appropriately indirect (Tronto, 1993). That being said, there are other relationships, say between the engineers and users, that could mutually benefit from a more caring connection. Tronto is careful to remind that moral qualities of care should not be treated as virtues, as this tends towards an individualist conception of moral development where one can cultivate virtues on their own (2013, p. 35). Care ethics, on the other hand, commits to a relational ontology (Section 3) wherein caring qualities emerge only through connection with others. For example, a robotics engineer cannot take up responsibility for workers in the silo of the lab. Responsibility is instead something that develops through cycles of asking, listening, and acting that make up a relationship of care.

### Embodiment at Work

As claimed in Section 3.1, taking care of people’s bodies is an essential part of caring for people’s well-being. In the context of work, I argue that caring for bodies should occur at two levels: one, workers’ basic bodily needs must be met, and, two, the role of HRI in shaping one’s sense of self should be considered.

When applying care ethics to a robotized distribution center or factory, one of the first concerns is whether or not the basic bodily needs of eating, drinking, resting, urinating, and defecating have been met. If not, there can be little progress on more abstract concepts common to the future of work discussions, like meaningfulness or agency. This may seem so obvious it is not worth discussing, but ethical discourse can mistakenly skip over people’s most base requirements. While the capability approach takes basic needs seriously (Roebyns, 2017; Nussbaum 2013), even in regards to technology design (Oosterlaken, 2012; van den Hoven, 2012), it says little about the caring relationships that enable people to take up the resources they need. The two theories are not mutually exclusive and are even compatible (Tronto 2013), but for the purposes of this paper let us delve further into the ways embodied relationality of HRI might affect a worker’s well-being.

Directly focusing on people’s bodies is crucial when discussing robotics at work because new technical capabilities open new possibilities for embodied interactions between workers and robots. A robot may affect a person’s embodiment directly with its physicality, and indirectly with its cultural and political meaning. In the first instance, a robot’s integration on the work floor requires some changes to the environment and to the workflow of employees. This might bring immediate relief and also prevent long-term injuries, thereby contributing to well-being. But, working alongside a robot is not necessarily without its physical challenges of its own. People are asked to work at a specific speed, twist and grab at a certain height, or stay out of the robots’ way. These changes may lead to new injuries or physical burdens (Harwell, 2019).

In addition to the extra physical toll potentially caused by immediate interaction between humans and robots, one’s embodiment at work can be shaped by personal, social, and/or cultural pressures. As discussed in Section 4.1, the use of robots is supported by a complex network of people. Coeckelbergh (2021) argues that this means all robots are embedded in social and cultural practices and therefore take on a socio-relational meaning, i.e., both humans and robots co-shape each other’s meaning, both culturally and socially. To further explore this thought at the bodily level, I draw on the work of feminist phenomenologist, Iris Marion Young, who provides a useful description of how oppression can be internalized and taken up in the body’s movements. In *Throwing Like a Girl*, she responds to Erwin Straus’ assessment that young girls toss balls a shorter distance than boys because of a biological difference (1980, 27). Using Straus’ descriptions, where boys use their whole body to throw while girls just use one arm, Young comes to another conclusion: girls have an “inhibited intentionality,” where their sense of “I can” is stunted by a self-imposed “I cannot” (36). This limiting schism is not the result of biology, but is taken on by girls because they live in a society that treats them like objects, does not empower them to take ownership over their lives, and is frequently unsafe for them (Young, 1980). Thus, they move in stunted ways that lack wholeness and freedom.

Social roles and norms shape people’s embodiment when working with robots, too. Dobrosovestnova and Hannibal (2020) also draw from the feminist theory of identity to show how being employed beside service robots can perpetuate the pressure to take up gendered and racialized norms at work. A service robot that is unerringly chipper and helpful can make human employees feel the need to maintain a similar outward show. Dobrosovestnova and Hannibal (2020) argue that women are particularly burdened by this performance because they are already expected to be more accommodating than men. This pressure is taken up at the level of the body, through welcoming smiles and empathetic nods of the head. Of course, humans are not always smiling or patient, so this effort to maintain robotic levels of consistency distances a worker from her humanity and leads to self-alienation (Dobrosovestnova & Hannibal, 2020, p. 153).

The pressure to mimic robotic traits can be observed in industrial and retail settings, as well. When placed beside robots, workers report the pressure to work faster and more consistently over long periods of time, which is something that humans do not find easy to do (Harwell, 2019; Fletcher & Webb, 2017). In some troubling reports from Amazon warehouses, workers say they wear diapers so that they do not waste time going to the bathroom (Harwell, 2019). As said in Section 2.4, this example is illustrative of an organization that values profit over well-being, a culture of consumerism that expects next-day delivery, and shows the need for proper labor laws. It can also tell us something about how larger social, political, and economic pressures are taken up in a person’s physical form and movement. Like with the girls described by Straus and Young, workers are contorting their bodies (e.g., ignoring injuries or the need to urinate) under the weight of job insecurity. As Coeckelbergh explains, a single robot can take on a larger social meaning (2021), which here might be one’s own replaceability and potential financial strain. In response, people try to compete or match robotic levels of consistency at the cost of their humanity at the most base and bodily level. Certainly, this stands in stark contrast to narratives claiming that robots will help people do more “human activities” (Section 2.2).

It is also possible that the robots could help workers embody the “I can” that Marion Young describes in the boys’ movements. For example, an exoskeleton might enable a worker to lift heavier objects with freedom and ease, allowing them to make full-body movements with confidence. However, if the exoskeleton is not coupled with a caring infrastructure at multiple levels (e.g., strong labor policies that ensure adequate working conditions, managers that prioritize workers’ well-being, and empathetic co-workers), employees might be expected to lift more items faster during their shift, leaving them depleted. A care ethics analysis takes the complexity of embodied experience into account by recognizing that well-being is not an achievement of the mind, but an embodied and relational experience.

### Emotions at Work

As with the body, care ethics includes emotions as a source of knowledge when caring for one’s well-being (Chatzidakis et al., 2020; Gilligan, 1982; Tronto, 1993). Dominant Western thinking has pit emotion against rational thought, long associating it with a lack of control, the body, and femininity, all of which are deemed secondary at best (Tronto, 1993). While care itself need not be sentimental (Tronto, 1993), care ethics deems emotions as important contributors to well-being and critical to understanding if a person is cared for.

The dismissal of emotion extends into the philosophy of technology, as emotions are not often taken seriously in decision-making about new technologies (Roeser, 2018; Roeser & Pesch, 2015) nor when deploying robots in the workplace (Fletcher & Webb, 2017; van Wynsberghe, Ley & Roeser, 2021). Concern for the emotional effects of robot use is often reserved for care robots, especially ones used by children or vulnerable populations. However, the introduction of robots into a distribution center or store is not without its emotional impact (Fletcher & Webb, 2017; Guendelsberger, 2019; Harwell, 2019). When seeking to understand the effects of robots in the workplace, a care ethics perspective would take emotions as essential information and starting point for further inquiry.

Emotions, too, should be understood in terms of their multi-level relationality. A robot can affect a person’s emotions through direct and immediate interactions, say by disrupting the workflow and giving rise to frustration. At the organizational level, a person may feel pleased to work for a company that integrated robotics in a helpful way. At the societal level, a person may be fearful of a new robot because robots are often depicted as evil or manipulative in movies and TV shows. Embodiment plays a role here, too: someone might feel happy to work with a robot that does the heavy lifting. The emotion of happiness indicates that the robot is being deployed in such a way that meets the needs of the worker, contributing to their well-being at work.

Workers’ emotions may also be directly affected by forming a relationship with the robot itself. As robots become more adaptive and dynamic, the more likely people are to attribute a mind and intentionality to the machine (Nyholm, 2020, 137–138). Materiality affects this relationship as well, as soft robotics invite people into more tactile connection with robots, leading to stronger emotional attachments to the machines (Arnold and Scheutz, 2017). The emotional relationship workers form towards robots in industry or retail is rarely a key focus in the literature, but is nonetheless a critical aspect of someone’s well-being at work (Fletcher and Webb, 2017; van Wynsberghe, Ley and Roeser, 2021).

Robots’ social, cultural, and political meanings also affect people’s emotional responses to robots. If people do not feel secure in their employment, the robot can come to represent personal insecurity. Emotions like anger, fear, or jealousy may be the result. Not only do these strong feelings block a person’s sense of well-being in the moment, but further investigation may reveal that they do not feel secure, recognized, or valued. Taking emotions seriously provides an opportunity to learn what people need to be cared for.

Emotions have another role to play in the creation of caring work environments. The Care Collective reminds that caring is often imbued with complex emotions (2020, p. 28)—feeling burdened by the need to care can lead to resentment, love for another might keep one at a job they hate, and fury at a government’s inaction can give a person the courage to fight for change. Emotions drive care and they inhibit it, therefore should be identified and understood when seeking to create caring infrastructures at work.

## Conclusions: Towards a Caring Future of Work

I have focused on the present as a transitory moment, where robots have begun to step beyond barriers and work alongside humans, but have not yet taken up any of the more far-off visions of the media, corporations, or some academics. The future of work, then, is still malleable. It is possible to make it more caring, though it would require n immense change in relationships at individual, organizational and political levels. Using a care ethics analysis can help towards this end, though requires further development and refinement in this particular application.

This paper begins this exploratory work by arguing that, first, caring relationships and care gaps should be mapped out. Then, if there is a commitment to foster attentiveness, responsibility, competence responsiveness, and solidarity in these dynamics, a more caring future of work might emerge. I argue that this approach also brings forward two oft-overlooked aspects of relationships in industrial and retail work: embodiment and emotions. I propose that embodiment should be understood beyond safety, by looking at how people’s bodies take up societal and economic pressures when placed beside robots. And lastly, I argue that workers’ emotions are essential to understanding how well-being at work might be promoted.

Since care has a long history of being seen as feminine, weak, and intimate, a care ethics approach might be quickly dismissed from the industrial or retail settings that seem to align with more masculine qualities like strength and productivity. But these traditional dichotomies only serve old prejudices, and blind from the fundamental and unshakeable dependance humans have on care. Care ethics is not delicate or impractical. Since the 1980s, philosophers taking this approach have argued that caring without action is merely an intention or sentiment. This may be nice to feel, but is not enough to support human well-being. Care ethics involves discerning how people are doing and what they need, then responding to these needs with practical action. Certainly, this direct caring approach would be helpful in shaping a future of work that supports people’s well-being.

## Data Availability

N/A.

## Footnotes

1 This is a hotly debated topic and engaging in it fully would stray away too from the focus of this paper. If you are interested in the topic of robot agency, personhood, and rights, I suggest beginning with these resources: Amanda Sharkey, “Can robots be responsible moral agents? And why should we care?” (2016) and with Noel Sharkey “Granny and the robots: ethical issues in robot care for the elderly” (2012); Aimee van Wynsberghe and Scott Robbins, “Critiquing the Reasons for Making Artificial Moral Agents” (2019); David Gunkel, *Robot Rights* (2018); Coeckelbergh, “Robot rights? Towards a social-relational justification of moral consideration” (2010).

2 Value-sensitive design is a useful methodological resource when seeking to discern the full network of people involved in a technology (Friedman & Hendry, 2019).
